# The Influence of Dietary Fiber (β-Glucan) on the Beneficial Effects of Phenolic Compounds from Chokeberry After Simulated Digestion In Vitro

**DOI:** 10.3390/molecules30163356

**Published:** 2025-08-12

**Authors:** Lidija Jakobek, Ivica Strelec, Petra Matić

**Affiliations:** Faculty of Food Technology Osijek, Josip Juraj Strossmayer University of Osijek, Franje Kuhača 18, 31000 Osijek, Croatia; ivica.strelec@ptfos.hr (I.S.); petra.matic@ptfos.hr (P.M.)

**Keywords:** antiradical activity, α-amylase, α-glucosidase, diabetes, dietary habits

## Abstract

Bioactive phenolic compounds released in the digestive tract have the potential to mitigate various diseases. However, they can be affected by dietary fibers. Our aim was to study the influence of β-glucan (dietary fiber) on the antiradical activity of phenolic compounds from chokeberry and its inhibition of α-amylase and α-glucosidase after digestion. These beneficial activities, helpful in many health issues connected to the digestive tract, depend on the constituents of food, such as dietary fibers, that surround these compounds and are not completely elucidated. Simulated digestion of chokeberry with or without the presence of β-glucan was conducted in vitro. The released phenolic compounds (RP-HPLC method), the antiradical activity (DPPH method), and the inhibition of α-amylase and α-glucosidase were determined after digestion. Chokeberry after gastric and intestinal digestion showed antiradical activity, and after intestinal digestion, it inhibited α-amylase and α-glucosidase. B-glucan decreased the amount of total phenolic compounds released (1800 to 1761 mg kg^−1^ fw) and bioaccessibility (60 to 59%) in the stomach (*p* < 0.05) and small intestine (1738 to 1637 mg kg^−1^ fw, 58 to 55%) (*p* < 0.05), decreased the antiradical activity, and weakened the enzyme inhibition. Principal component analysis clustered the released phenolic compounds and beneficial effects according to digestion with or without added β-glucan, confirming the influence of β-glucan on beneficial effects. Chokeberry polyphenols kept their beneficial effects in the stomach and small intestine in the presence of dietary fiber, which allows us to suggest that they show bioactivities even in the presence of other food constituents.

## 1. Introduction

The gastrointestinal tract is the first system in the body exposed to food and is often considered the first system to experience the beneficial effects of food. One such beneficial effect is the inhibition of digestive enzymes by compounds from food, which can be helpful for diabetes. Diabetes is a disease that causes serious problems for a large number of people and impairs their quality of life. Patients with diabetes suffer from increased blood glucose levels or hyperglycemia. This is especially important after a meal, when the blood glucose level can significantly increase. One way to alleviate the symptoms of diabetes is to inhibit the enzymes responsible for the breakdown of polysaccharides into glucose in the digestive system, which might slow down or decrease the absorption of glucose in the blood [1,2]. In particular, α-amylase accelerates the degradation of polysaccharides into smaller sugar molecules, while α-glucosidase plays important roles in accelerating the final degradation of polysaccharides and liberating glucose. Compounds from food that inhibit those enzymes in the digestive tract have potential beneficial effects. Phenolic compounds from fruits such as black mulberry [1], black chokeberry [3], or sweet and sour cherries [2] inhibit α-amylase [1,2,3] and α-glucosidase activity [1,2], a fact that can be used in the prevention and treatment of diabetes [1].

Another cause for concern in the gastrointestinal tract involves free radicals or oxygen-containing free radicals (reactive oxygen species, ROS). ROS are active oxygen-centered molecules and can be divided into free radical ROS (superoxides, hydroxyl radicals, peroxyl radicals, alkoxyl radicals) and non-radical ROS (oxygen, ozone, hydrogen peroxide, chloramines, hypochlorous acid, or organic hydroperoxides [4] such as lipid hydroperoxides). They can be generated in the digestive tract [4,5] or introduced by a meal of a high-fat food, processed food, or fried food [5,6]. In fact, it is considered that the intestine is an important source of ROS [4,7]. ROS can enhance the lipid peroxidation process in the gastrointestinal tract. This is a process of oxygen degradation of lipids that is initiated when ROS attack lipids. The result of lipid peroxidation is the formation of lipid hydroperoxides which can break down to various reactive species such as reactive aldehydes, ketones, or epoxides [5]. Once absorbed in the intestine, those reactive species can interact with important biological molecules, causing damage. High levels of ROS or greater lipid peroxidation might be connected to malignant diseases, diabetes [8], or other gastrointestinal diseases [7]. One way to mitigate the effects of free radicals is to capture free radicals within the digestive system. Antioxidants can inhibit the generation of ROS [4] and thus inhibit lipid peroxidation in the gastrointestinal tract [9,10]. Consequently, antioxidants can mitigate the effects of free radicals and lipid peroxidation products in the gastrointestinal tract.

In numerous studies, phenolic compounds have been shown to be beneficial in both the aforementioned problems—they possess antiradical activity [1,11] and they can inhibit the activity of digestive enzymes [1,2]. A fruit with high amounts of phenolic compounds is the chokeberry (*Aronia melanocarpa*) [3,12,13,14,15,16,17,18,19]. However, phenolic compounds undergo numerous changes in the digestive system [20], and their effects after release in the digestive system are not fully elucidated. The release of phenolic compounds from the food matrix begins in the mouth. In the stomach, the release continues, but the released amounts are still lower than the amounts present in fresh fruit [20,21,22]. A low pH value in the stomach allows phenolic compounds to not degrade much in the stomach. In the case of chokeberry, it has been shown that main groups of phenolic compounds such as anthocyanins, flavonols, phenolic acids, and flavan-3-ols are released in the stomach; however, the amounts are still lower than the amounts naturally present in the fruit [20,22], while the amount of some phenolic acids increases [20,21]. Upon reaching the small intestine, with increased pH, the amounts of phenolic compounds decrease, and changes in phenolic compounds may occur. Examples of these changes in chokeberry phenolic compounds include the change in the form of anthocyanins and the isomerization of chlorogenic acid into cryptochlorogenic acid [21]. The beneficial effects of phenolic compounds within the digestive system should be considered in accordance with the composition of released phenolic compounds and changes occurring in phenolic compound molecules.

In addition to the amounts released from food and the forms present in the digestive tract, the effects of phenolic compounds should be considered in accordance with other food constituents ingested with them. Among such compounds are dietary fibers. Dietary fibers are not digested in the upper digestive tract but are broken down by the enzymes of the gut microbiota in the lower digestive tract [23]. Accordingly, their presence in the stomach and small intestine might have an impact on the bioactivity of phenolic compounds. The effects of dietary fibers on the bioactivity of phenolic compounds are not clear. β-glucan is a soluble dietary fiber found in foods such as barley and oats and is also used as a dietary supplement due to its positive effects on human health [24]. Chemically, β-glucans are non-starch polysaccharides, and those from cereals are composed of D-glucopyranosyl units connected with β—(1→3) and β—(1→4) linkages, with molecular weights from 31–2700 × 10^3^ (barley) or 65–3100 × 10^3^ g mol^−1^ (oats) [24]. They are soluble in water due to having many OH groups [24]. β-glucan can interact with phenolic compounds and potentially affect their bioactivities in the digestive system.

The aim of this work was to study the antiradical activity of, and the inhibition of α-amylase and α-glucosidase by, phenolic compounds from chokeberry (*Aronia melanocarpa*) after simulated gastrointestinal digestion in vitro and the influence of β-glucan on these beneficial effects. The digestion of chokeberry or chokeberry with added β-glucan in the stomach and small intestine was conducted in vitro, and the phenolic compounds released were characterized with reversed-phase high-performance liquid chromatography (RP-HPLC) to determine their bioaccessibility. Samples after digestion were studied for their beneficial effects (antiradical activity in the stomach and antiradical activity and the inhibition of α-amylase and α-glucosidase in the small intestine). To find the differences between the phenolic compounds released in digestion with or without the presence of β-glucan, and differences in their beneficial effects, various statistical tests were applied to the results, such as a post hoc Tukey test, a *t*-test and principal component analysis. Studies on the influence of β-glucan on the beneficial effects of phenolic compounds in the gastrointestinal tract can indicate how the food matrix impacts these beneficial effects. The results might suggest how to consume phenolic compounds to achieve the best effects in the gastrointestinal tract—between meals when there is no interaction with the food matrix, or during the meal, when phenolic compounds are surrounded by other food constituents. To the best of our knowledge, the influence of β-glucan on bioactivities of phenolic compounds from chokeberry after digestion has not been studied till now.

## 2. Results

### 2.1. Identification of Phenolic Compounds in Chokeberry

Figure S1 shows a chromatogram of chokeberry before digestion with identified phenolic compounds. Maxima of their UV/Vis spectra are shown in Table S1. The compounds identified are phenolic acids (neochlorogenic acid, chlorogenic acid), flavonols (rutinoside, galactoside, glucoside of quercetin), anthocyanins (galactoside, glucoside, arabinoside and xyloside of cyanidin) and flavan-3-ols ((−)-epicatechin). Neochlorogenic and chlorogenic acid have a typical maximum at 326 nm with a shoulder at 298 nm. Flavonols showed two maxima, at 256 and 354 nm, while anthocyanins were characterized with maxima at 278–282 nm and 516 nm, and (−)-epicatechin had a maximum at 280 nm. The identification agrees with the data published in the literature [14,15,16].

Figure S2 shows a chromatogram with identified phenolic compounds obtained after gastric and small intestinal digestion. The same compounds present in the chokeberry before digestion were identified after gastric digestion. All phenolic compounds were identified in the small intestine as well, with the exception of cryptochlorogenic acid, which was identified only in the small intestine. In the small intestine, chlorogenic acid (5-caffeoylquinic acid) and neochlorogenic acid (3-caffeoylquinic acid) can isomerize into cryptochlorogenic acid (4-caffeoylquinic acid) due to alkaline conditions, as already reported in the literature [21]. The isomerization occurs when the caffeoyl group detaches from the hydroxyl group at position 5 or 3 and attaches to the hydroxyl group at the position 4 of the quinic acid. The isomerization of chlorogenic acid or neochlorogenic acid in the small intestine probably resulted in the formation of the cryptochlorogenic acid in this study.

### 2.2. Amounts and Distribution of Phenolic Compounds in Digestion

The amounts of phenolic compounds in fruits before digestion and after the gastric and intestinal phases of digestion are shown in Table 1. Chokeberry contained 2040 mg per kg of fresh weight (fw) of phenolic acids before digestion, followed by 523 mg kg^−1^ fw of anthocyanins, 319 mg kg^−1^ fw of flavonols, and 109 mg kg^−1^ fw of flavan-3-ols, which is a total of 2990 mg kg^−1^ fw. The amounts agree with the literature [12,14,15,16,21]. The amounts of phenolic compounds released in gastric digestion were significantly lower than before digestion (*p* < 0.05) (1800 and 1761 mg kg^−1^ fw, without or with added β-glucan, respectively). The same can be seen for all phenolic groups; the amounts were significantly lower than those before digestion (*p* < 0.05). The decrease in the content of phenolic compounds from chokeberry after gastric digestion was already reported in earlier studies [21,25]. After digestion in the small intestine, total phenolic compounds additionally decreased to 1738 and 1637 mg kg^−1^ fw (without or with added β-glucan, respectively), but not significantly in comparison to the stomach. However, the decrease was significant compared to the amount before digestion. The behavior of individual phenolic groups in the small intestine varied in comparison to the stomach. The amounts of anthocyanins significantly decreased, while the amounts of flavonols significantly increased (*p* < 0.05) in the small intestine in comparison to the amounts in the stomach. Anthocyanins change their structure with changes in pH. At lower pH values such as the pH of the stomach, they exist in the form of flavylium cations. With the increase in pH in the small intestine (pH 7), they can change their chemical form to a cabinol pseudobase [20]. This might be the reason for their decrease in the small intestine. In addition, the formation of anthocyanin adducts might have led to the reduction in the anthocyanin amount, as reported in an earlier study [21]. On the other hand, flavonols showed stability in the small intestine, and their amount increased, probably due to their additional release. Phenolic acids were present in the small intestine in a high percentage and amount; however, they were not stable. Chlorogenic acid can isomerize into cryptochlorogenic acid at the pH of the small intestine, as mentioned earlier [21]. Accordingly, the formation of cryptochlorogenic acid after small intestinal digestion might be explained by the isomerization of chlorogenic and neochlorogenic acid into cryptochlorogenic acid.

**Figure 1:**
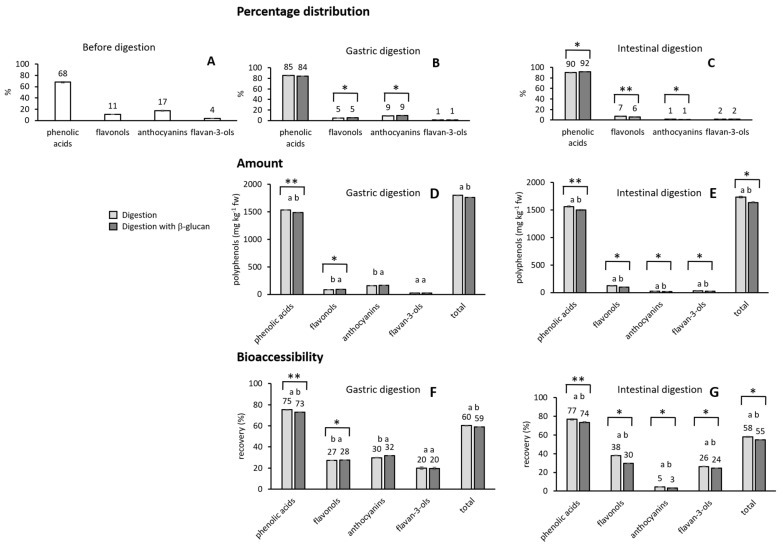
Percentage distribution of phenolic groups in chokeberry (**A**) before digestion and (**B**) after gastric and (**C**) intestinal digestion. The amount of phenolic compounds in chokeberry after digestion (**D**) in the stomach and (**E**) in the small intestine. Bioaccessibility of phenolic compounds after the digestion of chokeberry (**F**) in the stomach and (**G**) in the small intestine. Light color—without β-glucan, dark color—with added β-glucan. The data with different letters are significantly different according to the post hoc Tukey test (*p* < 0.05). Also *, ** mark significant difference with a *t*-test with *p* < 0.05, *p* < 0.01, respectively.

Percentage distributions of phenolic compounds are shown in Figure 1A–C. Before digestion, chokeberry contained 68% of phenolic acids as a major phenolic group, 11% flavonols, 17% anthocyanins, and 4% flavan-3-ols. According to [13], different varieties of chokeberries contained 9.3 to 13.7% of anthocyanins, which is similar to our results, 44.3 to 59.4% of proanthocyanidins, and 29.2 to 44.6% of other polyphenols. The sample of chokeberry in this study contained a higher percentage of phenolic acids than reported earlier [13]. Phenolic acids dominated in the stomach as well, occupying 84–85% of the total phenolic compounds, which is higher than before digestion. Favonols occupied 5%, anthocyanins 9%, and flavan-3-ols 1%. Phenolic acids were the major phenolic group in the small intestine as well, occupying 90 to 92% of the total amount, while flavonols occupied 6 to 7%, anthocyanins 1%, and flavan-3-ols 2%.

**Figure 2:**
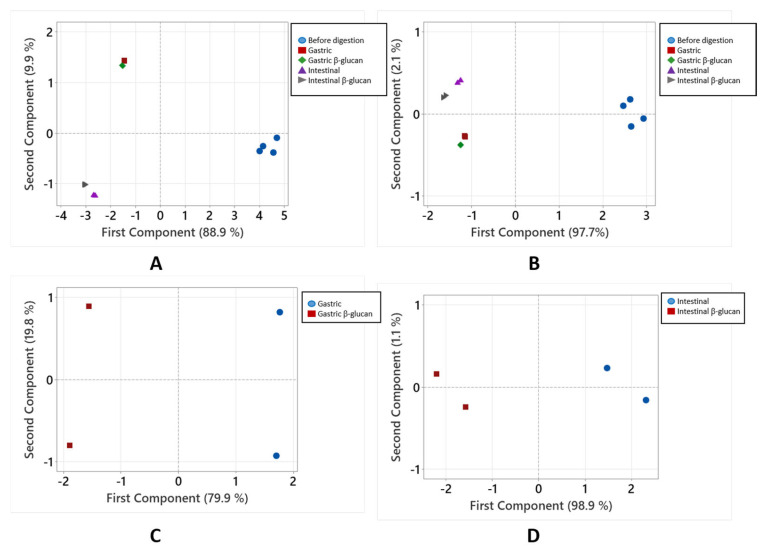
Principal component analysis of the phenolic compounds. Analyzed data are (**A**) the amounts of all individual phenolic compounds before digestion and after gastric and intestinal digestion, (**B**) total amounts of phenolic groups before digestion and after gastric and intestinal digestion, (**C**) the amounts of phenolic groups after gastric digestion without and with β-glucan, and (**D**) the amounts of phenolic groups after intestinal digestion without and with β-glucan.

The overall results concerning the amounts suggest a decrease in phenolic compounds in the stomach and additionally in the small intestine, in comparison to the amount before digestion. The content decreases in the small intestine as the result of the environment having a higher pH value. To confirm the differences between phenolic compounds in chokeberry and those released in gastric and small intestinal digestion, principal component analysis (PCA) was conducted on the data (Figure 2A,B). PCA clearly showed clustering of the individual phenolic compounds (Figure 2A) and phenolic groups (Figure 2B) according to amounts before digestion and after gastric and intestinal digestion. The first principal component explained 88.9% (Figure 2A) and 97.7% (Figure 2B) of the total variance. This suggests that the amounts before digestion and those released in the gastric phase and in the small intestinal phase are different.

### 2.3. The Influence of β-Glucan on the Release of Phenolic Compounds

Some differences in the release of phenolic compounds with the addition of β-glucan are visible in Table 1, where a post hoc Tukey test was used to analyze all the values before and after digestion. In gastric digestion, the amount of total phenolic acids decreased, while that of flavonols and anthocyanins increased, when β-glucan was present in digestion, in comparison to gastric digestion without β-glucan. In the small intestine, the addition of β-glucan decreased the amount of all phenolic groups and total phenolic compounds. However, most of those differences were not significant. To further study the differences with the addition of β-glucan, results were analyzed with additional statistical tests, including a post hoc Tukey test and a *t*-test (Figure 1D,E). This time, only the amounts in the specific digestion phase without or with β-glucan were analyzed. Now, it became visible that in the stomach the decrease in phenolic acids and total phenolic compounds with the addition of β-glucan is significant, while the amounts of flavonols and anthocyanins significantly increased (*p* < 0.05) (Figure 1D). In the small intestine (Figure 1E), all phenolic groups and total phenolic compounds significantly decreased when β-glucan was added in digestion (*p* < 0.05).

Figure 1F,G show the bioaccessibility of phenolic compounds. From 59 to 60% of total phenolic compounds were accessible for absorption in the stomach, and 55 to 58% were accessible for absorption in the small intestine. The bioaccessibility of phenolic acids and total phenolic compounds significantly decreased in the stomach (*p* < 0.05) with the addition of β-glucan, while the bioaccessiblity of anthocyanins and flavonols increased (*p* < 0.05) (Figure 1F). In the small intestine (Figure 1G), the bioaccessibility of all phenolic groups and total phenolic compounds significantly decreased with the addition of β-glucan (*p* < 0.05).

To confirm the results of the influence of β-glucan on the release of phenolic compounds, principal component analysis (PCA) was used to analyze the amounts released in digestion conducted with or without added β-glucan (Figure 2C,D). The PCA in the gastric phase shows grouping according to digestion without or with β-glucan (Figure 2C) with the first principal component accounting for 79.9% of the total variability. The same can be seen for the intestinal phase of digestion (Figure 2D), where the first principal component explained 98.9% of the total variance. The grouping confirms that there is a separation between amounts of released compounds depending on the presence of β-glucan.

It can be suggested that β-glucan can adsorb phenolic compounds, which leads to a lower amount of them in their free form in the digestive tract. The adsorption of phenolic compounds onto β-glucan was already reported in the literature [26,27]. In our earlier studies, we described the adsorption of phenolic compounds from aronia [28,29] or apples [30,31] onto β-glucan and the adsorption of individual phenolic compounds such as procyanidins [32], flavan-3-ols, dihydrochalcones, anthocyanins [33], and phenolic acids [34] onto β-glucan. Phenolic compounds might bind to β-glucan with non-covalent bonds such as Van der Waals forces or H bonds. β-glucan has the potential to affect the release of phenolic compounds in the stomach and small intestine, which can affect their accessibility for absorption and possibly other bioactivities. On the other hand, β-glucan can carry phenolic compounds in higher amounts to the colon, where they can be released and show potential bioactivities.

### 2.4. Antiradical Activity of Phenolic Compounds in Digestion

Phenolic compounds from chokeberry showed antiradical activity in many earlier studies [12,13]. The aim of this study was to determine the antiradical activity of chokeberry after digestion with the DPPH method. The results were expressed as percentages of inhibition of DPPH radicals and as EC_50_ values, which are the μmols of total phenolic compounds needed to scavenge 50% of free DPPH radicals (Table 2). Higher EC_50_ value represents lower antiradical activity. Phenolic compounds after gastric digestion showed antiradical activity (0.0594 and 0.0695 μmol of phenolic compounds, without or with added β-glucan, respectively). The antiradical activity increased after intestinal digestion (0.0169 and 0.0165 μmol phenolic compounds, without or with added β-glucan, respectively). This increase was statistically significant (*p* < 0.05). To confirm the influence of phenolic compounds released in digestion on the antiradical activity, the percentage inhibition of DPPH radicals was correlated with the amount of phenolic compounds. The correlation was positive and high (R^2^ was 0.98, 0.98, 0.99, and 0.99 for gastric digestion, gastric digestion with added β-glucan, intestinal digestion, and intestinal digestion with added β-glucan, respectively), which can lead to the suggestion that phenolic compounds in digestion have the ability to scavenge free radicals. The antiradical activity of phenolic compounds after digestion agrees with the study of Köpsel et al. [25]. They studied the antiradical activity of chokeberry juice and chokeberry juice with added cow’s milk or oat or soya drink, with the DPPH method. Those samples showed antiradical activity after digestion, similar to this study. Moreover, β-glucan might have affected the antiradical activity in this study. EC_50_ values point to weaker antiradical activity after gastric digestion with β-glucan (17% different) in comparison to the same digestion without added β-glucan. However, those differences were not significant and were shown only in gastric digestion. When the antiradical activity was studied with the DPPH assay, in samples after the digestion of chokeberry juice or chokeberry juice with added oat drink and soya drink, the antiradical activity in samples with added oat and soya drinks was lower in comparison to chokeberry juice [25], similarly to this study.

Dietary fibers might have the ability to interfere with the antiradical activity of phenolic compounds. However, the effects of dietary fibers on the phenolic compounds in the digestive tract need to be studied further to confirm the results. The viscosity of β-glucan is dependent on the β-glucan concentration. Consequently, different concentrations of β-glucan might affect the antiradical activity in a different way, which was not studied here.

### 2.5. The Inhibition of α-Amylase and α-Glucosidase

The inhibition of α-amylase and α-glucosidase was studied for samples after digestion in the small intestine. Percentages of inhibition were calculated, as well as amounts of phenolic compounds needed to inhibit the enzyme activity by 50% (IC_50_ value) (Table 2). Lower IC_50_ values represent higher inhibition activity.

Chokeberry inhibited the activity of α-amylase (IC_50_ 0.0378 and 0.0415 μmol phenolic compounds, in digestion without or with added β-glucan, respectively). The amounts of phenolic compounds present in digestion positively correlated with the percentage of inhibition (R^2^ values were 0.97 and 0.92 for digestion without or with the addition of β-glucan, respectively). In other words, the higher the amounts of released phenolic compounds in digestion, the stronger the inhibition of enzyme activity. In earlier studies, phenolic compounds such as phenolic acids [35,36], anthocyanins from berries [37,38], or procyanidins form lychee seeds [11] inhibited α-amylase activity, which agrees with this study. All of these mentioned phenolic compounds bonded to α-amylase [11,35,36,37,38], with the benzene ring with OH groups more effective for bonding [35,38]. Finally, bonding exhibited changes in the spatial conformation of α-amylase [38]. Moreover, phenolic compounds altered the hydrophobic environment of amino acids, and they likely bonded to the enzyme on a single binding site, which induced conformational changes in α-amylase [11]. Some studies investigated the inhibition of α-amylase after the gastrointestinal digestion of phenolic compounds [39]. The phenolic compounds of *Chaenomeles japonica* (the main constituents were procyanidins) and *Hippophaë rhamnoides* (the main constituents were isorhamnetin derivatives) after gastrointestinal digestion inhibited the activity of α-amylase [39], which agrees with this study. It was again suggested that the inhibition was related to the direct interaction of phenolic compounds with enzymes [39]. In accordance with the earlier studies mentioned, it might be suggested that phenolic compounds from chokeberry after gastrointestinal digestion inhibited α-amylase due to their interaction with α-amylase, which might have caused the conformational changes in the enzyme and ultimately its lower activity. It should be mentioned that even an experiment with α-amylase inhibition can somewhat influence inhibition. When starch (substrate) and phenolic compounds are incubated together during the experiment, before adding the enzyme, bonding between the substrate (starch) and phenolic compounds can affect the final inhibition of the enzyme [36,37]. In this study, samples after digestion were first incubated with the starch, which might have affected the final inhibition percentage. Finally, interactions between starch and phenolic compounds and between enzymes and phenolic compounds could lead to the weaker activity of a-amylase after intestinal digestion.

Chokeberry samples after intestinal digestion inhibited the activity of α-glucosidase (IC_50_ 0.0177 and 0.0225 μmol phenolic compounds in digestion without or with added β-glucan, respectively). The amounts of phenolic compounds positively correlated with the inhibition percentage (R^2^ was 0.79 and 0.82 for samples without or with added β-glucan). It can be suggested that the higher amounts of phenolic compounds might have caused the stronger inhibition of the enzyme activity. In addition, phenolic compounds from chokeberry were stronger inhibitors of α-glucosidase activity than acarbose, a known drug for type 2 diabetes, used to slow down the digestion of carbohydrates. In earlier studies, phenolic acids [35] and anthocyanins from blackcurrant [38] inhibited α-glucosidase activity. Again, those phenolic compounds bonded to α-glucosidase [35,38], which caused conformational changes in the enzyme [38] and ultimately the inhibition of enzyme activity. In accordance with earlier studies, it might be suggested that phenolic compounds from chokeberry after gastrointestinal digestion inhibited α-glucosidase due to the interaction with the enzyme, which might have caused the conformational changes in the enzyme and ultimately its lower activity.

Many fruit extracts rich in phenolic compounds inhibited the activity of α-amylase and α-glucosidase in earlier studies [1,2,3,19], including chokeberry [3], which agrees with this study. Different phenolic groups were connected with the stronger inhibition activity in extracts; however, the results are still inconclusive. In four different fractions extracted from black mulberry, the anthocyanin fraction showed stronger inhibitory activity against α-amylase and α-glucosidase [1]. When different varieties of sour and sweet cherries inhibited α-amylase and α-glucosidase, the inhibition was not correlated with the amount of phenolic compounds [2]. It was mentioned that the specific type (structure of ring) might be more relevant to the inhibitory effects than the amount [2]. In chokeberry, the most potent inhibitor of α-amylase from the group of phenolic acids was chlorogenic acid, and in the group of anthocyanins, it was cyanidin-3-glucoside [3]. Pure phenolic compounds were also studied for their inhibition of digestive enzymes in earlier studies. When pure phenolic acids such as chlorogenic acid were tested for the inhibition of α-amylase, they inhibited α-amylase [40,41,42]. However, some studies have mentioned that phenolic acids are poor inhibitors of α-amylase [42]. As chlorogenic acid is the ester of caffeic acid and quinic acid, it was found that the caffeoyl moiety decreased the inhibition of chlorogenic acid [40]. Pure anthocyanins (cyanidin-3-glucoside, cyanidin-3,5 diglucoside, cyanidin-3-rutinoside, and peonidin-3-glucoside) inhibited the α-amylase activity as well [43], and amongst them, cyanidin-3-glucoside showed the strongest inhibition, followed by cyanidin-3-rutinoside [43]. Cyanidin-3-glucoside inhibited α-glucosidase as well [44]. Various flavonols [45] and quercetin [46] inhibited α-amylase. According to those earlier studies, some suggestions can be made for this study. Phenolic acids were the major constituents of samples after the intestinal digestion of chokeberry, and they could have contributed to the inhibitory activity, together with anthocyanins and flavonols. Additional studies are needed to see which phenolic compounds from chokeberry are more important for the inhibition of enzymes after digestion. In addition, anthocyanins in the form of a carbinol pseudobase should be studied in the future.

B-glucan might have affected the inhibition. When β-glucan was present in digestion, the inhibition of α-amylase was 9.8% weaker (0.0415 μmol of phenolic compounds) in comparison to the inhibition without β-glucan (0.0378 μmol of polyphenols). This difference was not statistically significant. Similarly, when β-glucan was present in digestion, phenolic compounds showed 27% weaker inhibition of the α-glucosidase activity (0.0225 μmol), in comparison to the sample without β-glucan (0.0177 μmol), which was also not statistically significant. The effects of different concentrations of β-glucan were not studied here. Since different concentrations of β-glucan might contribute to the inhibition of digestive enzymes in a different way, the influence of β-glucan concentration needs to be studied further.

### 2.6. The Influence of β-Glucan on Bioactivities in the Digestive Tract

**Figure 3:**
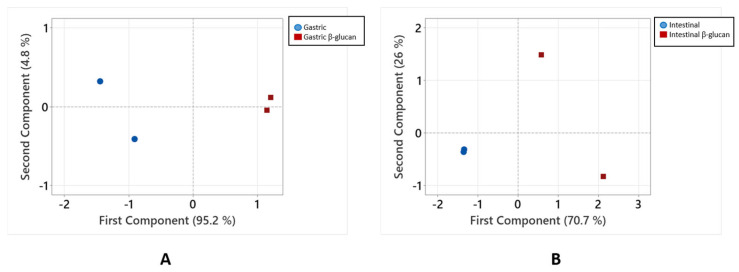
Principal component analysis of the phenolic compounds and their bioactivities after digestion without or with added β-glucan. Analyzed data include (**A**) total phenolics and antiradical activity in the gastric digestion, (**B**) total phenolics, antiradical activity, and the inhibition of α-amylase and α-glucosidase in the intestinal digestion.

To confirm whether β-glucan affects the bioactivities of polyphenols from chokeberry after digestion, principal component analysis was used to analyze the data. Figure 3 shows the results of the analysis of total phenolics and their antiradical activity in the stomach (Figure 3A) and total phenolics, antiradical activity, and inhibition of α-amylase and α-glucosidase in the small intestine (Figure 3B). Released compounds and their bioactivities are clustered according to whether digestion was conducted without or with added β-glucan. In the gastric digestion, the first principal component separated the data according to the presence of β-glucan and explained 95.2% of the total variance (Figure 3A). In the small intestinal digestion, the first principal component explained 70.7% of the total variance. It can be suggested that the addition of β-glucan in digestion changes the amount of released phenolic compounds as well as their bioactivities.

## 3. Discussion

The digestive tract is the first system exposed to food and to all compounds generated by digestion. Some of the processes taking place in the digestive tract and the compounds generated there affect various diseases that burden modern society, such as diabetes, ulcerative colitis, gastric cancer, or inflammatory bowel disease [4,7]. Compounds such as polyphenols, reported in many earlier studies as bioactive, have a high potential to show bioactivities directly in the digestive tract, before they are absorbed. Phenolic compounds from chokeberry released in the stomach have a similar composition to fresh fruit. In the stomach, they consisted of 84 to 85% phenolic acids, 9% anthocyanins, 5% flavonols, and 1% flavan-3-ols. Consequently, it can be expected that phenolic compounds in the stomach might have the same bioactivities as those shown for fresh, native fruit through earlier studies. Indeed, in this study, after the digestion in the stomach, released phenolic compounds showed antiradical activity. On the other hand, the environment of the small intestine affected the released compounds. Chlorogenic and neochlorogenic acid isomerized into cryptochlorogenic acid, and anthocyanins changed their form, as we already reported in our earlier studies [31,47]. However, the major compounds were still phenolic acids (90–92%), followed by flavonols (6 to 7%), flavan-3-ols (2%), and anthocyanins (1%). Due to some changes in the composition of phenolic compounds, the expected bioactivities need to be additionally studied. Still, in this study, phenolic compounds released in the small intestine from chokeberry showed antiradical activity and the inhibition of α-amylase and α-glucosidase.

Food rich in lipids goes through lipid peroxidation in the stomach, a chain process, initiated by free radicals, which results in the generation of various lipid radicals and lipid peroxides. Products of the breakdown of lipid peroxides can be cytotoxic [5], and they can be absorbed in the intestine. Several studies connected them and other reactive oxygen species to many chronic diseases [4,5,7,8]. Phenolic compounds from chokeberry that showed antiradical activity can be helpful in scavenging free radicals and accordingly helpful in the prevention of the mentioned processes. This positive bioactivity might be of importance in the stomach as well as in the small intestine.

Hyperglycemia is the state of a higher concentration of glucose in the blood. Especially after a meal, elevated concentrations of glucose in the blood can be a serious problem for diabetic patients. Since chokeberry inhibited the activity of α-amylase and α-glucosidase in the small intestine, it might be studied further for its helpfulness in alleviating hyperglycemia and type 2 diabetes.

B-glucan affected the beneficial effects of phenolic compounds shown in the stomach and small intestine. It weakened their ability to scavenge free radicals and to inhibit enzymes. Those effects might be explained by the adsorption of phenolic compounds onto the β-glucan surface, which is a bigger molecule. This leads to less free phenolic compounds in the digestive tract that would otherwise show bioactivities. However, β-glucan itself showed some positive bioactivities. β-glucan can show antiradical activity with a DPPH assay [48,49]. Due to possible bonding of phenolic compounds to β-glucans during digestion, the antiradical activity of β-glucan might have changed in a way to weaken its ability to scavenge free radicals. Consequently, the weakened ability of β-glucan to scavenge free radicals and the lower amount of free phenolic compounds finally showed weaker antiradical activity in samples after digestion. Furthermore, according to earlier studies, β-glucan itself inhibited α-amylase activity [50,51]. β-glucan inhibits α-amylase by noncompetitive inhibition, and it was suggested that it interacts with α-amylase with hydrogen bonding and Van der Waals forces, which leads to the structural adjustment of α-amlyase and to inhibition [51]. In addition, β-glucan might form a physical barrier between starch and α-amylase, leading to its inhibition of α-amylase activity [51]. Even though β-glucan can inhibit α-amylase itself, in this study, its role in adsorbing phenolic compounds might be more prominent. β-glucan might have adsorbed phenolic compounds on its surface, which prevented its bonding to α-amylase and led to weaker inhibition of α-amylase. Consequently, the lower amount of free phenolic compounds and weaker ability of β-glucan to act as an inhibitor led to the overall weaker inhibition. Due to its ability to adsorb phenolic compounds, β-glucan might have lowered the inhibition of α-glucosidase as well. To confirm this, the inhibition of α-amylase by phenolic compounds and β-glucan together in digestion needs further studies.

It is often questioned when and how to use food to increase the beneficial effects of some of the food compounds. Dietary habits are as important in beneficial effects as the activity of individual compounds. In other words, whether food rich in phenolic compounds is consumed alone between meals or with other foods during a meal can be crucial in specific beneficial effects in the body. This study showed that phenolic compounds from chokeberry kept their potential for antiradical activity and enzyme inhibition even with the presence of β-glucan (dietary fiber). This can lead to the conclusion that even if chokeberries (phenolic compounds) are consumed in the diet together with other foods (food constituents), chokeberry should still show antiradical and inhibitory activity. Similarly, Stanisavljević et al. [52] concluded that chokeberry phenolics undergo transformation during digestion, but they still keep their antioxidant role in the presence of the food matrix. Further studies are necessary to determine the proper dietary habits that can be helpful for patients with diabetes or other health issues, which will include recommendations on whether to consume chokeberries alone between meals, after a meal, or during a meal with other foods.

Future studies should explore the influence of different concentrations of β-glucan/dietary fibers on the bioactivities of phenolic compounds, the possible formation of a complex β-glucan phenolic compound and its activity, the influence of other dietary fibers and phenolic compounds on other bioactivities in the digestive tract, and the influence of various food matrix on the bioactivities of phenolic compounds. Such studies will help in finding answers on how and when to use chokeberry in the diet.

## 4. Materials and Methods

### 4.1. Chemicals

Basic chemicals were purchased from Gram mol d.o.o. (Zagreb, Croatia) (potassium chloride, potassium dihydrogen phosphate, sodium hydrogen carbonate, calcium chloride, magnesium chloride, potassium sodium tartarate tetrahydrate), Kemika (Zagreb, Croatia) (ammonium carbonate), Carlo Erba Reagents (Val de Reuil, France) (sodium chloride), Fluka (Buchs, Switzerland) (ortho-phosphoric acid 85%), and J.T. Baker (Gliwice, Poland) (methanol). Acarbose (98%) was obtained from TCI Co., Ltd. (Tokyo, Japan), and 3,5-dinitrosalicylic acid was obtained from Thermo Fisher Scientific (Waltham, MA, USA). Standards of phenolic compounds were purchased from Extrasynthese (Genay, France) (cyanidin-3-galactoside chloride, and cyanidin-3-glucoside chloride and quercetin-3-galactoside) and Sigma-Aldrich (St. Louis, MO, USA) ((−)-epicatechin, quercetin-3-rutinoside; quercetin-3-glucoside; neochlorogenic acid, chlorogenic acid, cryptochlorogenic acid). Enzymes were also purchased from Sigma-Aldrich (St. Louis, MO, USA) (α-amylase A3176, 13 U/mg, pepsin P7000, 632 U/mg, α-glucosidase G 5003 100UN, pancreatin P7545, 8 USP), as well as bile salt, β-glucan from barley, 2,2-diphenyl-1-picryl-hydrazil (DPPH), and 4-nitrophenyl α-D-glucopyranoside.

### 4.2. Preparation of Solutions for the Simulated Digestion

Stock solutions of electrolytes were prepared ((KCl, KH_2_PO_4,_ (NH_4_)_2_CO_3_ 0.5 M), (NaHCO_3_ 1 M), (MgCl_2_ 0.15 M), (NaCl 2 M), (CaCl_2_ 0.3 M)). Simulated salivary fluid electrolyte solution (SSF), simulated gastric fluid electrolyte solution (SGF), and simulated intestinal fluid electrolyte solution (SIF) were prepared by pipetting appropriate volumes of the stock solutions of electrolytes in volumetric flasks. The final concentrations in simulated solutions were expressed in mmol l^−1^ (SSF—18.875 KCl, 4.625 KH_2_PO_4_, 17 NaHCO_3_, 0.056 MgCl_2_, and 0.06 mmol L^−1^ (NH_4_)_2_CO_3_; SGF—8.625 KCl, 1.125 KH_2_PO_4_, 31.25 NaHCO_3_, 0.15 MgCl_2_, 0.625 (NH_4_)_2_CO_3_, and 59 mmol L^−1^ NaCl; the pH of SGF was then adjusted to 3 with 1 M HCl, SIF—8.5 KCl, 1 KH_2_PO_4_, 106.25 NaHCO_3_, 0.4125 MgCl_2_, and 48 mmol L^−1^ NaCl, and the pH of SIF was adjusted to 7 with 1 M HCl). α-amylase (1000 mg L^−1^) was prepared in SSF, pepsin (31,660.61 mg L^−1^) in SGF, and pancreatin (8000 mg L^−1^) in SIF. Bile salts were prepared in SIF (25,000 mg L^−1^).

### 4.3. Chokeberry Sample

Chokeberry (*Aronia melanocarpa*) was harvested in an orchard in Orahovica (Croatia). According to earlier studies, the amount of dietary fibers in fresh chokeberry can be 5.6% [53]. The chokeberry fruit (100 g) was dried in a food dehydrator at room temperature using a flow of air (KYS-328A, Delimano, Guandong Kangye Electric Appliances Co., Foshan City, China) up to a point where 63 g of water was removed, leaving 37 g of dried fruit material with, according to the literature, 5.6 g of dietary fibers (15.14% in dry material). The dried fruit material was ground in a coffee grinder and then stored in vacuum-sealed bags before the analysis.

### 4.4. The Extraction of Phenolic Compounds from Chokeberry

A sample of dry chokeberry (0.06 g) was weighed in a plastic tube in which 1.5 mL of 80% methanol in water was added. Phenolic compounds were extracted by placing the tube in an ultrasonic bath (RK-100, Bandelin electronic GmbH, Berlin, Germany) for 15 min. The tube was then centrifuged (Eppendorf Minispin, Eppendorf, Hamburg, Germany) for 5 min (10,000 rpm) to separate the extract from the residue. The residue was extracted again in 0.5 mL 80% methanol using the same procedure. Next, the residue was extracted three more times by the same procedure but using a different solution, 0.55 mL 0.1% HCl. All collected extracts were combined, filtered with a 0.2 μm PTFE syringe filter, and analyzed with an RP-HPLC method (reversed-phase high-performance liquid chromatography).

Phenolic compounds from the residue were additionally extracted with an enzyme-assisted extraction. Distilled water (1.05 mL) was pipetted in the tube with the residue, and bile salt (60 μL), pancreatin (30 μL), and pepsin (15 μL) were added. The tube was placed in a dry block thermostat (Bio TDB-100, Biosan, Riga, Latvia) for 2 h at 37 °C and then in an ice bath. The mixture was centrifuged (5 min, 10,000 rpm) to separate the extract from the residue. The extract was filtered through the 0.2 μm PTFE syringe filter and analyzed with the RP-HPLC. The amounts of phenolic compounds after two types of extraction (the chemical and enzyme assisted extraction) were added together to represent the amount of phenolic compounds before digestion.

### 4.5. Simulated Digestion

A sample of chokeberry after the gastric digestion was prepared by conducting both salivary and subsequent gastric digestion according to [54]. Salivary digestion was conducted by adding dried chokeberry (0.06 g) into a plastic tube, together with 175 μL of SSF, 48.8 μL of H_2_O, 1.25 μL of CaCl_2_, and 25 μL of α-amylase. The tube was placed in the dry block thermostat for 2 min at 37 °C. The gastric digestion continued by adding 375 μL of SGF, 14.8 μL of H_2_O, 0.25 μL of CaCl_2_, 10 μL of HCl (1 M), and 100 μL of pepsin. After 2 h in the dry block thermostat at 37 °C, the mixture was placed in an ice bath for 5 min and centrifuged for 5 min (10,000 rpm), and the liquid sample was separated from the residue. The described procedure of digestion was repeated three times with new dried chokeberry samples. Liquid samples after three gastric digestions were combined into one sample.

A sample of chokeberry after the small intestine digestion was prepared by conducting salivary and gastric digestion as already described and then by continuing with small intestine digestion. After a sample of chokeberry went through salivary and gastric digestion, 550 μL of SIF, 180.5 μL of H_2_O, 2 μL of CaCl_2_ (0.3 M), 7.5 μL of NaOH (1 M), 250 μL of pancreatin, and 10 μL of bile salt were added to the mixture. The reaction mixture was placed in the dry block thermostat (37 °C) for 2 h. The mixture was placed in the ice bath for 5 min and centrifuged for 5 min (10,000 rpm), and the liquid sample was separated from the residue. The procedure of digestion was repeated four times with new dried chokeberry samples. Liquid samples after four consecutive small intestinal digestions were combined into one sample.

The liquid samples obtained after gastric and small intestinal digestion were placed in a water bath (LSB Aqua Pro, Grant Instruments Ltd., Shepreth, Cambridgeshire, UK) at 70 °C for 15 min to inactivate enzymes and then filtrated through a 0.2 μm nylon syringe filter. Filtrated samples were used for the analysis of antiradical activity and the inhibition of enzymes. Aliquots of filtrated samples (0.6 mL) were filtrated one more time with a 0.2 μm PTFE syringe filter and analyzed with RP-HPLC. All liquid samples and residues were stored at −18 °C for one day before the analysis.

The simulated digestion of chokeberry with added β-glucan was conducted according to the same procedure. The amount of dried chokeberry in each experiment was again 0.06 g (with 15.14% or 0.0091 g of dietary fiber). The amount of β-glucan to be added in each experiment was chosen as 20% of dietary fibers (0.0091 g) already present in 0.06 g of dried chokeberry, which is 0.0018 g. Accordingly, each reaction mixture had 0.06 g of dried chokeberry and 0.002 g of β-glucan. In addition, a blank of simulated digestion that contained water instead of chokeberry was conducted.

The bioaccessibility was calculated as a recovery.

(1) $$\left(recovery\right)\left(\%\right)\;=\;\frac{\gamma_{digestion\;phase}(mg\;{kg}^{-1})}{\gamma_{before\;digestion}(mg\;{kg}^{-1})}100$$

*γ*_digestion phase_ is the amount of a phenolic compound after the gastric or intestinal digestion phase (mg kg^−1^ fresh weight (fw)), and *γ*_before digestion_ is the amount of a phenolic compound before digestion determined with chemical and enzyme-assisted extraction (mg kg^−1^ fw).

### 4.6. RP-HPLC Analysis of Phenolic Compounds

Phenolic compounds were identified and quantified by using an HPLC system (1260 Infinity II, with a quaternary pump, a PDA detector, and a vial sampler (Agilent technology, Santa Clara, CA, USA). Phenolic compounds were separated on a Poroshell 120 EC C-18 column (4.6 × 100 mm, 2.7 μm) and a Poroshell 120 EC-C18 4.6 mm guard column (Agilent technology, Santa Clara, CA, USA) with a mobile phase A (0.5% H_3_PO_4_) and mobile phase B (100% acetonitrile). The flow rate was set at 0.5 mL min^−1^. The gradient conditions included a gradually increasing B phase, from 5% in 0 min, to 11% in 5 min, 15% in 7.5 min, 17.5% in 17.5 min, 20% in 20 min, 30% in 30 min, and 70% in 32 min; it continued at 70% at 34 min and decreased to 5% in 36 and 38 min. Compounds were identified by comparing UV/Vis spectra of peaks in samples with those of standards. Standards of various concentrations were analyzed to create calibration curves, which were used for the quantification. Calibration curves were linear (r^2^ = 0.9942 to 1). The amounts of phenolic compounds were expressed as mg kg^−1^ fresh weight (fw) or in μmol μL^−1^. Cyanidin-3-arabinoside and cyanidin-3-xyloside were quantified by using the calibration curve of cyaniding-3-glucoside. Precision was determined by calculating the coefficient of variation of multiple measurements of the same sample (0.02 to 14.99%).

### 4.7. Antiradical Activity

The antiradical activity of samples after gastric and small intestinal digestion was determined with a DPPH method (2-2-diphenyl-1-picrylhydrazyl): 200 μL of DPPH (1 mM in methanol) was mixed with 2800 μL of methanol, and the absorbance *A*_DPPH_ was read using a UV/Vis spectrophotometer (UV 1280, Shimadzu, Kyoto, Japan) at 517 nm against a blank. Reaction mixtures were prepared by adding 200 μL of DPPH solution (1 mM) and various volumes of samples after gastric (10, 20, 50, 80 and 100 μL) or intestinal digestion (5, 10, 15, 20 and 30 μL) into cuvettes, and the rest of the volume was methanol up to the final volume of 3000 μL. The absorbance *A*_sample_ was determined against the blank (contained the same volumes of samples, and the rest of the volume was methanol) after 1, 2, 3, 4, 5, 10, 15, 20, and 30 min. The inhibition of DPPH radicals was calculated according to the following equation:

(2) $$DPPH\;inhibition\left(\%\right)\;=\;\frac{A_{DPPH}\;-\;A_{sample}}{A_{DPPH}}\;\times \;100$$

Percentages of DPPH radical inhibition after 5 min were plotted against the amount of phenolic compounds (μmol) in the reaction mixture, and the following equation was obtained: y = ax + b (y = % inhibition, x = μmol of phenolic compounds). This equation was used to calculate the amount of polyphenols that inhibited the DPPH radical by 50% (EC_50_).

### 4.8. α-Amylase Inhibition

Potassium sodium tartarate tetrahydrate (5.3 M in 2 M NaOH) and 3,5-dinitrosalicylic acid (96 mM in water) were prepared. A color reagent was prepared by mixing warm water (30 mL), potassium sodium tartarate tetrahydrate (20 mL), and 3,5-dinitrosalicylic acid (50 mL). α-amylase (2 U/mL) was prepared in a phosphate buffer (6.9 pH, 0.02 M), and starch (1%) in the water. Reaction mixtures were prepared in four glass tubes. The first tube represented a control (a buffer pH 6.9, starch, the sample after the blank digestion, and α-amylase), the second was the control blank (buffer pH 6.9, starch, sample after the blank digestion), the third represented the inhibition (buffer pH 6.9, starch, liquid sample after digestion, α-amylase), and the fourth was the inhibition blank (buffer pH 6.9, starch, liquid sample after digestion). Final volumes in tubes were always 500 μL. The volumes of starch and α-amylase were always 240 and 150 μL, respectively. Volumes of samples were 5 to 60 μL. The mentioned reagents were pipetted in tubes according to the following procedure: after pipetting the buffer, starch, and sample after digestion in tubes, the tubes were placed in an incubator (IN 30 Memmert, Schwabach, Germany) at 37 °C for 10 min. Then, α-amylase was added, and the mixtures were again placed in the incubator for 10 min at 37 °C. Then, 250 μL color reagent was added, and all tubes were placed in the water bath (100 °C, 10 min) and cooled down. An additional 2250 μL of water was added in each tube so that the final volume was always 3000 μL, and the absorbance was measured at 540 nm by using the UV/Vis spectrophotometer.

The absorbance of the control (*A_control_*) was measured against its blank, and the absorbance of the sample inhibitor (*A_sample_*) was measured against its blank. The *inhibition* was calculated according to the following equation:

(3) $$\%\;inhibition\;=\;\frac{A_{control}\;-\;A_{sample}}{A_{control}}\;\times \;100$$

By comparing the absorbance of a sample after the digestion of chokeberry (*A_sample_*) with the absorbance of the sample after the blank digestion (*A_control_*), the influence of the digestion solutions on the inhibition was eliminated. The percent inhibition was plotted against the concentration of phenolic compounds in the sample after digestion, and the curve was used to calculate the concentration of inhibitor that caused 50% inhibition of α-amylase (IC_50_).

### 4.9. α-Glucosidase Inhibition

Solutions needed for the experiments were prepared (phosphate buffer pH 6.9, 0.1 M; α-glucosidase 4 U/mL in water; substrate 4-nitrophenyl α-D-glucopyranoside (pNGP) 5 mM in buffer pH 6.9; and acarbose 1 mM in buffer pH 6.9). Reaction mixtures were prepared in four glass tubes; the first was the control (buffer pH 6.9, pNGP, α-glucosidase), the second was the control blank (buffer pH 6.9, pNGP), the third was the inhibition (buffer pH 6.9, pNGP, sample after digestion, α-glucosidase), and the fourth was the inhibition blank (buffer pH 6.9, pNGP, sample after digestion). Final volumes in tubes were always 3000 μL. Volumes of pNGP and α-glucosidase were always 250 μL, and volumes of samples were 5 to 200 μL. After pipetting a buffer, pNGP, and a sample after digestion in tubes, the tubes were placed in the incubator at 37 °C for 10 min. Then, α-glucosidase was added, and mixtures were again placed in the incubator for 17 min at 37 °C. The absorbance of the control (*A_control_*) was measured against its blank, and the absorbance of the inhibitor (*A_sample_*) was measured against its blank at 405 nm. The inhibition was calculated according to the following equation:

(4) $$\%\;inhibition\;=\;\frac{A_{control}\;-\;A_{sample}}{A_{control}}\;\times \;100$$

The percent *inhibition* was plotted against the concentration of phenolic compounds in samples (μmol), and the curve was used to calculate the concentration of phenolic compounds that causes 50% inhibition of α-glucosidase (IC_50_). The results were compared to acarbose, which was tested with the same procedure (volumes of acarbose 100, 200, and 400 μL).

A blank sample of digestion was also tested (the control contained a buffer pH 6.9, pNGP, a blank sample of the digestion, and α-glucosidase; the control blank contained a buffer pH 6.9, a blank sample of the digestion, and pNGP). However, it was determined that the blank sample of digestion did not have an influence on the inhibition since the absorbance of these samples was the same as absorbances when a blank sample of the digestion was not present in the mixture.

### 4.10. Statistical Analysis

Chemical and enzyme-assisted extractions were conducted two times, and each sample was measured using HPLC two times (*n* = 4). Gastric and intestinal phases of the digestion of chokeberry or chokeberry with added β-glucan were conducted once and analyzed using HPLC two times (*n* = 2). The antiradical activity was measured for various volumes of samples after digestion two times. The inhibition of enzymes was measured two times for various volumes of samples (*n* = 2). The results were expressed as mean ± standard deviation. The results were analyzed by using a post hoc Tukey test, *t*-test, and principal component analysis (Minitab LLC., State College, PA, USA).

## 5. Conclusions

Phenolic compounds were released from chokeberries in the gastric and intestinal phases of digestion. Released amounts were lower than the amount present in the fresh fruits, and they decreased from the gastric to the intestinal phase of digestion. Phenolic acids were the major constituents released in the stomach and in the small intestine. Samples after gastric and intestinal digestion showed antiradical activity, and samples after intestinal digestion inhibited α-amylase and α-glucosidase activity. Phenolic compounds correlated with the inhibition percentage. B-glucan affected digestion. It decreased the amount of released phenolic compounds in the stomach and small intestine, and it decreased the antiradical activity and the inhibition of enzymes, although this was not always statistically significant. In future studies, the influence of β-glucan concentration on the bioactivities of phenolic compounds should be studied in more detail due to changes in viscosity with changes in the concentration of β-glucan [24]. Moreover, future studies need to focus on the effects of other dietary fibers such as cellulose on the bioactivities of phenolic compounds. Cellulose is present in plant material and accordingly in everyday diets.

## Figures and Tables

**Table 1:** Amounts of phenolic compounds in chokeberry (mg kg^−1^ fw) before digestion and released during the digestion phases with or without β-glucan.

Phenolic Compounds	Before Digestion	Gastric Digestion		Gastric with β-Glucan		Intestinal Digestion		Intestinal with β-Glucan	
Phenolic acids									
neochlorogenic acid	1128.9 ±80.3 ^a^	970.3	±0.7 ^ab^	934.0	±0.6 ^b^	724.7	±6.3 ^c^	699.5	±4.5 ^c^
chlorogenic acid	910.8 ±68.3 ^a^	565.5	±0.2 ^b^	551.7	±0.7 ^b^	487.0	±5.9 ^b^	459.8	±7.8 ^b^
cryptochlorogenic acid						353.1	±2.2 ^a^	339.9	±1.0 ^b^
Total	2039.7 ±148.5 ^a^	1535.8	±0.9 ^b^	1485.8	±1.3 ^b^	1564.8	±14.3 ^b^	1499.3	±13.2 ^b^
Flavonols									
unknown	48.9 ±4.3 ^a^	13.8	±0.0 ^c^	14.1	±0.0 ^c^	24.6	±0.1 ^b^	23.1	±0.1 ^bc^
quercetin-3-rutinoside	63.8 ±1.6 ^a^	28.3	±0.0 ^c^	28.4	±0.0 ^c^	32.7	±0.4 ^b^	25.4	±0.2 ^c^
quercetin-3-galactoside	105.1 ±1.6 ^a^	21.7	±0.0 ^c^	23.2	±0.0 ^c^	31.1	±0.4 ^b^	20.6	±0.2 ^c^
quercetin-3-glucoside	101.5 ±1.3 ^a^	23.1	±0.1 ^c^	22.4	±0.0 ^c^	32.9	±0.4 ^b^	25.2	±0.0 ^c^
Total	319.3 ±8.7 ^a^	86.9	±0.1 ^c^	88.0	±0.1 ^c^	121.3	±1.2 ^b^	94.2	±0.5 ^c^
Anthocyanins									
cyanidin-3-galactoside	225.9 ±18.4 ^a^	69.1	±0.1 ^b^	80.9	±0.1 ^b^	19.1	±0.5 ^c^	15.9	±0.1 ^c^
cyanidin-3-glucoside	54.9 ±3.0 ^a^	19.4	±0.9 ^b^	16.6	±0.0 ^b^	0.6	±0.1 ^c^	0.3	±0.1 ^c^
cyanidin-3-arabinoside *	198.0 ±17.8 ^a^	54.2	±2.8 ^b^	56.7	±0.1 ^b^	3.4	±0.5 ^c^	0.8	±0.1 ^c^
cyanidin-3-xyloside *	43.9 ±3.3 ^a^	13.1	±0.0 ^b^	11.6	±0.1 ^b^	0.4	±0.4 ^c^	0.0	±0.0 ^c^
Total	522.7 ±42.5 ^a^	155.8	±3.9 ^b^	165.8	±0.2 ^b^	23.6	±1.5 ^c^	17.1	±0.2 ^c^
Flavan-3-ols									
(−)-epicatechin	108.6 ±8.7 ^a^	21.6	±1.3 ^b^	21.3	±1.3 ^b^	28.5	±0.5 ^b^	26.6	±0.4 ^b^
Total	108.6 ±8.7 ^a^	21.6	±1.3 ^b^	21.3	±1.3 ^b^	28.5	±0.5 ^b^	26.6	±0.4 ^b^
TOTAL	2990.3 ±208.4 ^a^	1800.2	±6.2 ^b^	1760.9	±3.0 ^bc^	1738.2	±17.6 ^bc^	1637.2	±14.4 ^c^

* tentatively identified. The data with different small case letters in the row are significantly different according to the post hoc Tukey test (*p* < 0.05).

**Table 2:** The antiradical activity and inhibition of enzymes α-amlyase and α-glucosidase of samples after the digestion of chokeberry in the stomach and small intestine.

	Antiradical Activity		Enzyme Inhibition			
	EC_50_		IC_50_ (α-Amylase)		IC_50_ (α-Glucosidase)	
	(μmol Polyphenols)		(μmol of Polyphenols)		(μmol of Polyphenols)	
Gastric	0.0594	±0.0040 ^bA^				
gastric + β-glucan	0.0695	±0.0003 ^aA^				
Intestinal	0.0169	±0.0002 ^cA^	0.0378	±0.0014 ^a^	0.0177	±0.0002 ^bA^
intestinal + β-glucan	0.0165	±0.0014 ^cA^	0.0415	±0.0027 ^a^	0.0225	±0.0009 ^bA^
Acarbose					0.3690	±0.0133 ^a^

The antiradical activity expressed after 5 min of reaction, the inhibition of α-amylase expressed after 10 min of reaction, and the inhibition of α-glucosidase expressed after 17 min of reaction. Lowercase letters in the same column mark the difference between values based on a post hoc Tukey test and *p* < 0.05. Uppercase letters in the same column mark the difference between values obtained for gastric digestion vs. gastric digestion + β-glucan or intestinal digestion vs. intestinal with β-glucan. The differences are based on a post hoc Tukey test and *p* < 0.05.

## Data Availability

The original contributions presented in the study are included in the article/Supplementary materials; further inquiries can be directed to the corresponding author.

## Supplementary Materials

The following supporting information can be downloaded at https://www.mdpi.com/article/10.3390/molecules30163356/s1, Figure S1: Chromatogram of chokeberry extract before digestion scanned at 280, 320, 360, and 510 nm with identified phenolic compounds. Peak identification: 1—neochlorogenic acid, 2—chlorogenic acid, 3—cyanidin-3-galactoside, 4—cyanidin-3-glucoside, 6—cyanidin-3-arabinoside*, 7—(−)-epicatechin, 8—cyanidin-3-xyloside*, 9—unknown flavonol, 10—quercetin-3-rutinoside, 11—quercetin-3-galactoside, 12—quercetin-3-glucoside (* tentatively identified); Figure S2: Chromatogram of chokeberry after chemical extraction, gastric and intestinal digestion scanned at 280 nm, 360 and 510 nm. Peak identification: 1—neochlorogenic acid, 2—chlorogenic acid, 3—cyanidin-3-galactoside, 4—cyanidin-3-glucoside, 5—cryptochlorogenic acid, 6—cyanidin-3-arabinoside*, 7—(−)-epicatechin, 8—cyanidin-3-xyloside*, 9—unknown flavonol, 10—quercetin-3-rutinoside, 11—quercetin-3-galactoside, 12—quercetin-3-glucoside (* tentatively identified); Table S1: Maxima of spectra of phenolic compounds from chokeberry.
