# Supplementary material for: The Influence of Dietary Fiber (β-Glucan) on the Beneficial Effects of Phenolic Compounds from Chokeberry After Simulated Digestion In Vitro
Source: Molecules. 2025 Aug 12;30(16):3356. doi: 10.3390/molecules30163356 (PMC12388337; doi:10.3390/molecules30163356)
Supplement: Supplementary file 1 [file molecules-30-03356-s001.zip › molecules-3753745-supplementary.pdf]

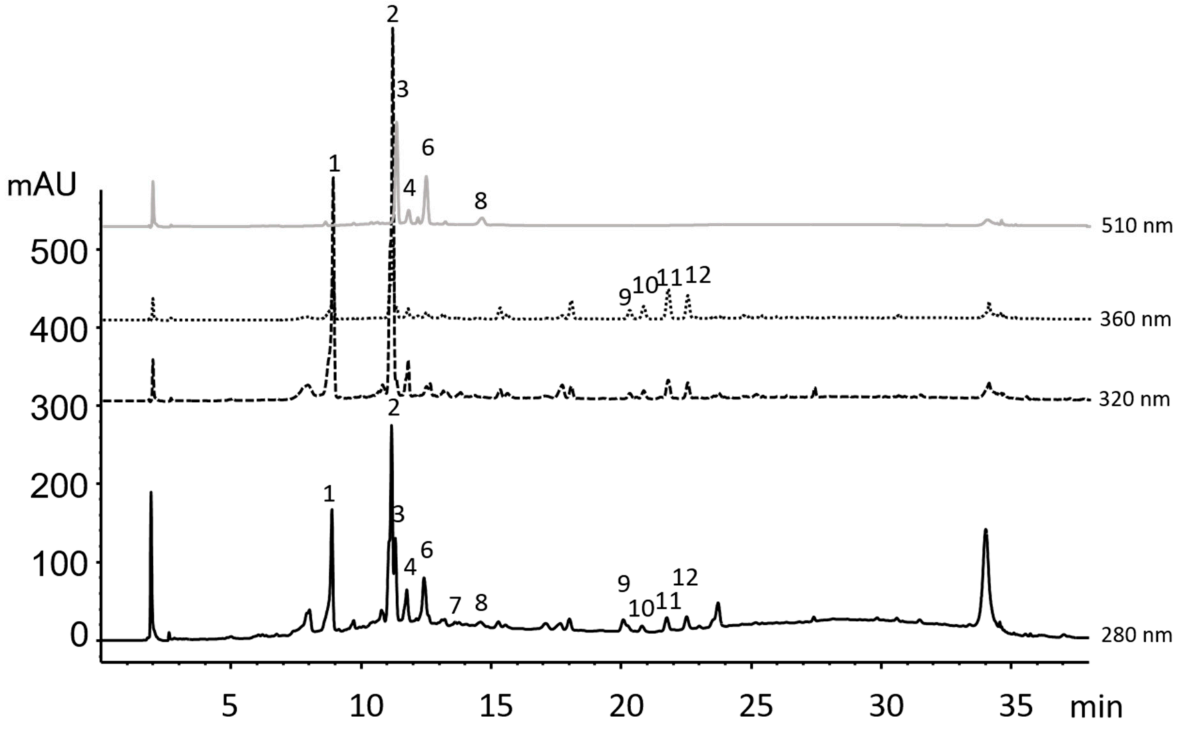

Figure S1. Chromatogram of chokeberry extract before digestion scanned at 280, 320, 360 and 510 nm with identified phenolic compounds. Peak identification: 1 – neochlorogenic acid, 2 – chlorogenic acid, 3 – cyanidin-3-galactoside, 4 – cyanidin-3-glucoside, 6 – cyanidin-3-arabinoside*, 7 – (-)-epicatechin, 8 – cyanidin-3-xyloside*, 9 – unknown flavonol, 10 – quercetin-3-rutinoside, 11 – quercetin-3-galactoside, 12 – quercetin-3-glucoside (* tentatively identified).

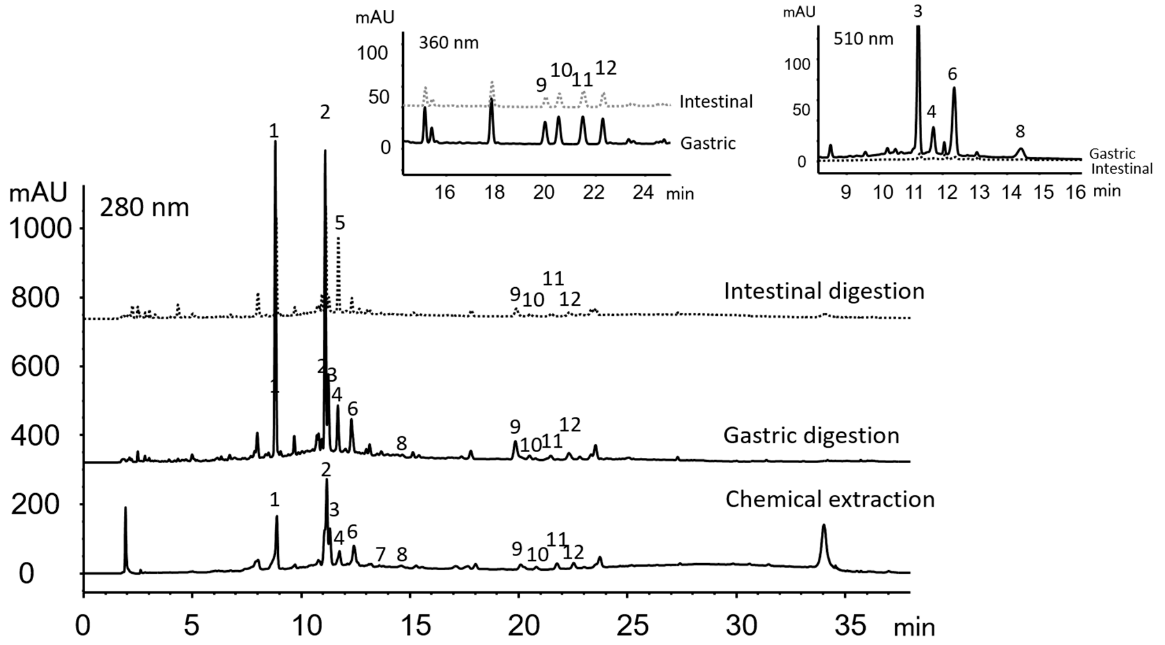

Figure S2. Chromatogram of chokeberry after chemical extraction, gastric and intestinal digestion scanned at 280 nm, 360 and 510 nm. Peak identification: 1 – neochlorogenic acid, 2 – chlorogenic acid, 3 – cyanidin-3-galactoside, 4 – cyanidin-3-glucoside, 5 – cryptochlorogenic acid, 6 – cyanidin-3-arabinoside*, 7 – (-)-epicatechin, 8 – cyanidin-3-xyloside*, 9 – unknown flavonol, 10 – quercetin-3-rutinoside, 11 – quercetin-3-galactoside, 12 – quercetin-3-glucoside (* tentatively identified).

Table S1. Maximums of spectra of phenolic compounds from chokeberry.

Peak identification	Phenolic compounds	Wavelength (nm)
1	neochlorogenic acid	sh 296, 326
2	chlorogenic acid	sh 298, 326
3	cyanidin-3-galactoside	280, 516
4	cyanidin-3-glucoside	280, 516
5	cryptochlorogenic acid	sh 298, 326
6	cyanidin-3-arabinoside*	282, 516
7	(-)-epicatechin	280
8	cyanidin-3-xyloside*	278, 516
9	Unknown	270, 354
10	quercetin-3-rutinoside	256, 354
11	quercetin-3-galactoside	256, 354
12	quercetin-3-glucoside	256, 354

sh – shoulder.

*Tentatively identified.
